# Pyridinium diaqua­bis­(methyl­enediphospho­nato-κ^2^ *O*,*O*′)chromate(III) tetra­hydrate

**DOI:** 10.1107/S1600536810028990

**Published:** 2010-07-24

**Authors:** Kina Van der Merwe, Hendrik G. Visser, J. A. Venter

**Affiliations:** a Department of Chemistry, University of the Free State, PO Box 339, Bloemfontein 9330, South Africa

## Abstract

In the title complex, (C_5_H_6_N)[Cr(CH_4_O_6_P_2_)_2_(H_2_O)_2_]·4H_2_O, the Cr^III^ atom, lying on an inversion centre, is coordinated by two bidentate methyl­ene diphospho­nate ligands and two water molecules in a distorted octa­hedral coordination geometry. The pyridinium cation is located on an inversion centre, with an N atom and a C atom sharing a position each at a half occupancy. A three-dimensional network is constructed by O—H⋯O, N—H⋯O and C—H⋯O hydrogen bonds between the pyridin­ium cation, complex anion and uncoordinated water mol­ecules.

## Related literature

For general background to metal-organic frameworks with diphospho­nic acids, see: Barthelet *et al.* (2002 ▶). For related structures, see: Byun *et al.* (2006 ▶); Suh *et al.* (1997 ▶); Van der Merwe *et al.* (2009 ▶); Visser *et al.* (2010 ▶).
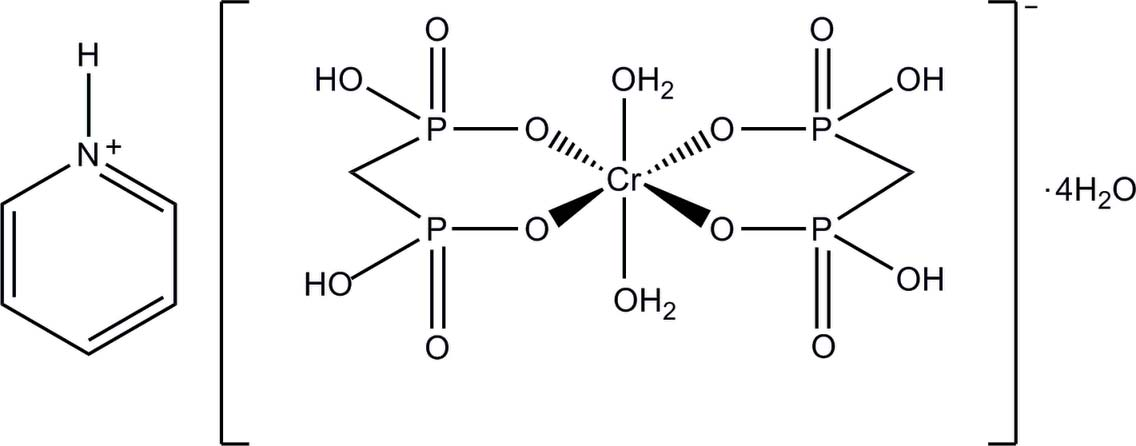


## Experimental

### 

#### Crystal data

(C_5_H_6_N)[Cr(CH_4_O_6_P_2_)_2_(H_2_O)_2_]·4H_2_O
*M* *_r_* = 588.17
Triclinic, 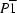

*a* = 7.206 (5) Å
*b* = 7.485 (5) Å
*c* = 10.984 (5) Å
α = 107.085 (5)°
β = 106.128 (5)°
γ = 94.496 (5)°
*V* = 535.7 (6) Å^3^
*Z* = 1
Mo *K*α radiation
μ = 0.92 mm^−1^
*T* = 100 K
0.22 × 0.16 × 0.08 mm

#### Data collection

Bruker APEXII CCD diffractometer
Absorption correction: multi-scan (*SADABS*; (Bruker, 2001 ▶) *T*_min_ = 0.843, *T*_max_ = 0.931
8784 measured reflections
2632 independent reflections
2483 reflections with *I* > 2σ(*I*)
*R*_int_ = 0.020

#### Refinement

*R*[*F* ^2^ > 2σ(*F* ^2^)] = 0.024
*wR*(*F* ^2^) = 0.070
*S* = 1.05
2632 reflections
179 parameters
16 restraints
H atoms treated by a mixture of independent and constrained refinement
Δρ_max_ = 0.47 e Å^−3^
Δρ_min_ = −0.62 e Å^−3^

### 

Data collection: *APEX2* (Bruker, 2007 ▶); cell refinement: *SAINT-Plus* (Bruker, 2007 ▶); data reduction: *SAINT-Plus*; program(s) used to solve structure: *SHELXTL* (Sheldrick, 2008 ▶); program(s) used to refine structure: *SHELXTL*; molecular graphics: *DIAMOND* (Brandenburg, 1999 ▶); software used to prepare material for publication: *SHELXTL*.

## Supplementary Material

Crystal structure: contains datablocks global, I. DOI: 10.1107/S1600536810028990/hy2333sup1.cif

Structure factors: contains datablocks I. DOI: 10.1107/S1600536810028990/hy2333Isup2.hkl

Additional supplementary materials:  crystallographic information; 3D view; checkCIF report

## Figures and Tables

**Table 1:** Selected bond lengths (Å)

Cr1—O1	1.991 (4)
Cr1—O2	1.956 (4)
Cr1—O7	1.964 (4)

**Table 2:** Hydrogen-bond geometry (Å, °)

*D*—H⋯*A*	*D*—H	H⋯*A*	*D*⋯*A*	*D*—H⋯*A*
C1—H4⋯O6^i^	0.97	2.49	3.346 (7)	147
C4—H4*A*⋯O9^ii^	0.93	2.16	2.93 (7)	140
N1—H1⋯O9^ii^	0.86	2.32	3.03 (5)	141
O1—H1*A*⋯O6^i^	0.80 (6)	1.83 (6)	2.634 (6)	176 (9)
O1—H1*B*⋯O4^iii^	0.83 (6)	1.87 (6)	2.704 (6)	177 (9)
O3—H3⋯O8^iv^	0.82	1.83	2.629 (6)	163
O5—H6⋯O4^ii^	0.83 (5)	1.80 (5)	2.619 (6)	175 (10)
O8—H7⋯O6^v^	0.83 (6)	1.86 (6)	2.687 (6)	171 (9)
O8—H8⋯O9	0.85 (7)	1.94 (8)	2.748 (7)	158 (11)
O9—H9*A*⋯O4	0.83 (6)	2.00 (6)	2.833 (6)	179 (10)
O9—H10⋯O8^vi^	0.84 (7)	1.99 (7)	2.820 (7)	174 (13)

Symmetry codes: (i) 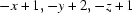; (ii) 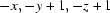; (iii) 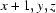; (iv) 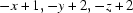; (v) 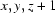; (vi) 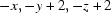.

**Fig. 1.**
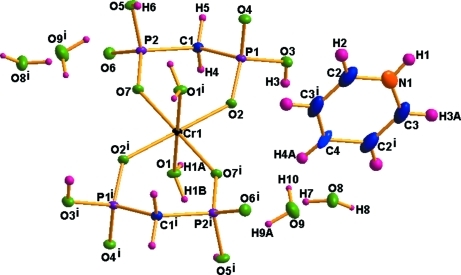
Molecular structure of the title compound. Displacement ellipsoids are drawn at the 50% probability level. [Symmetry code: (i) 1-x, 1-y, 1-z.]

## supplementary crystallographic information

### Comment

The title compound forms part of an ongoing study in our group involving methylene diphosphonate and its coordination to various metal cores. (Van der Merwe *et al.*, 2009; Visser *et al.*, 2010). Diphosphonic acids are useful for the synthesis of metal-organic frameworks exhibiting microporous properties (Barthelet *et al.*, 2002).

The Cr^III^ ion in the title complex is in a distorted octahedral environment (Fig. 1), with Cr—O bond distances ranging from 1.956 (4) to 1.991 (4) Å (Table 1). All the bond distances and angles are well within the normal range (Byun *et al.*, 2006; Suh *et al.*, 1997). The pyridinium cation is located on an inversion centre and an N atom and a C atom share a position at a half occupancy for each atom. A three-dimensional network is provided by numerous hydrogen bonds between the pyridinium cation, complex anion and uncoordinated water molecules (Table 2).

### Experimental

CrCl_3_.6H_2_O (0.092 g, 0.347 mmol) was dissolved in water (40 ml) and ammonium hydroxide was gradually added dropwise in order to precipitate Cr(III) hydroxide. Methylene diphosphonate (0.347 g, 2 mmol) was added to the Cr(OH)_3_ and water (40 ml). The reaction solution was heated on an oil bath for 5 h at 100°C, after which pyridine (10 ml) was added to the solution. Boiling H_2_O (30 ml) was added and the solution was centrifuged. Green crystals of the title compound crystallized from the filtrate after several days.

### Refinement

C-bound H atoms were positioned geometrically and refined as riding atoms, with C—H = 0.97 Å and *U*_iso_(H) = 1.2*U*_eq_(C). The H atoms attached to hydroxy groups and water molecules were located on a difference Fourier map and refined isotropically except H3, which was refined as riding, with O3—H3 = 0.82 Å and *U*_iso_(H3) = 1.5*U*_eq_(O3). A 50% positional disorder was assigned to N1 and C4, which share a position of the pyridine ring, as this provided the best fit of the data. Short C—C bond interactions, probably due to this disorder, are observed for the pyridinium cation.

### Crystal data

**Table:**

(C_5_H_6_N)[Cr(CH_4_O_6_P_2_)_2_(H_2_O)_2_]·4H_2_O	*Z* = 1
*M**_r_* = 588.17	*F*(000) = 303
Triclinic, *P*1	*D*_x_ = 1.823 Mg m^−^^3^
Hall symbol: -P 1	Mo *K*α radiation, λ = 0.71073 Å
*a* = 7.206 (5) Å	Cell parameters from 6300 reflections
*b* = 7.485 (5) Å	θ = 0.8–0.9°
*c* = 10.984 (5) Å	µ = 0.92 mm^−^^1^
α = 107.085 (5)°	*T* = 100 K
β = 106.128 (5)°	Cuboid, green
γ = 94.496 (5)°	0.22 × 0.16 × 0.08 mm
*V* = 535.7 (6) Å^3^	

### Data collection

**Table:**

Bruker APEXII CCD diffractometer	2483 reflections with *I* > 2σ(*I*)
φ and ω scans	*R*_int_ = 0.020
Absorption correction: multi-scan (*SADABS*; (Bruker, 2001)	θ_max_ = 28.3°, θ_min_ = 4.1°
*T*_min_ = 0.843, *T*_max_ = 0.931	*h* = −9→9
8784 measured reflections	*k* = −9→6
2632 independent reflections	*l* = −14→14

### Refinement

**Table:**

Refinement on *F*^2^	16 restraints
Least-squares matrix: full	H atoms treated by a mixture of independent and constrained refinement
*R*[*F*^2^ > 2σ(*F*^2^)] = 0.024	*w* = 1/[σ^2^(*F*_o_^2^) + (0.0342*P*)^2^ + 0.4874*P*] where *P* = (*F*_o_^2^ + 2*F*_c_^2^)/3
*wR*(*F*^2^) = 0.070	(Δ/σ)_max_ < 0.001
*S* = 1.05	Δρ_max_ = 0.47 e Å^−^^3^
2632 reflections	Δρ_min_ = −0.62 e Å^−^^3^
179 parameters	

### Fractional atomic coordinates and isotropic or equivalent isotropic displacement parameters (Å^2^)

**Table:**

	*x*	*y*	*z*	*U*_iso_*/*U*_eq_	Occ. (<1)
Cr1	0.5000	0.5000	0.5000	0.0082 (3)	
P2	0.2173 (2)	0.79359 (19)	0.41325 (14)	0.0086 (4)	
P1	0.29362 (19)	0.7622 (2)	0.69142 (14)	0.0085 (4)	
O2	0.4491 (6)	0.6457 (6)	0.6635 (4)	0.0110 (8)	
O5	−0.0069 (6)	0.7069 (6)	0.3539 (4)	0.0128 (8)	
O3	0.3700 (6)	0.8943 (6)	0.8418 (4)	0.0134 (8)	
H3	0.4774	0.9569	0.8570	0.020*	
O7	0.3373 (6)	0.6353 (6)	0.3947 (4)	0.0113 (8)	
O9	−0.0373 (8)	0.7358 (7)	0.8942 (5)	0.0235 (10)	
O1	0.7325 (6)	0.6953 (6)	0.5401 (4)	0.0121 (8)	
C1	0.2683 (8)	0.9145 (8)	0.5903 (6)	0.0108 (10)	
H4	0.3885	1.0063	0.6226	0.013*	
H5	0.1633	0.9841	0.6027	0.013*	
O4	0.0957 (6)	0.6494 (6)	0.6673 (4)	0.0118 (8)	
O8	0.2601 (7)	0.9720 (7)	1.1133 (5)	0.0198 (10)	
N1	0.381 (7)	0.479 (8)	0.070 (5)	0.028 (12)	0.50
H1	0.3025	0.4691	0.1145	0.034*	0.50
C4	0.381 (8)	0.466 (10)	0.078 (5)	0.024 (10)	0.50
H4A	0.3051	0.4405	0.1294	0.029*	0.50
C3	0.5612 (13)	0.5896 (11)	0.1367 (8)	0.0312 (17)	
H3A	0.6025	0.6454	0.2295	0.037*	
C2	0.3206 (12)	0.3837 (11)	−0.0627 (8)	0.0322 (17)	
H2	0.2008	0.3014	−0.1034	0.039*	
O6	0.2543 (6)	0.9385 (6)	0.3492 (4)	0.0133 (8)	
H8	0.193 (16)	0.882 (13)	1.042 (9)	0.06 (3)*	
H7	0.262 (14)	0.950 (13)	1.184 (7)	0.03 (2)*	
H1B	0.844 (10)	0.677 (12)	0.577 (8)	0.03 (2)*	
H1A	0.731 (13)	0.806 (9)	0.573 (8)	0.025*	
H6	−0.032 (11)	0.597 (8)	0.352 (9)	0.03 (3)*	
H9A	0.002 (13)	0.710 (13)	0.828 (8)	0.03 (2)*	
H10	−0.110 (17)	0.817 (16)	0.888 (14)	0.07 (4)*	

### Atomic displacement parameters (Å^2^)

**Table:**

	*U*^11^	*U*^22^	*U*^33^	*U*^12^	*U*^13^	*U*^23^
Cr1	0.0067 (6)	0.0081 (6)	0.0107 (6)	0.0020 (4)	0.0035 (4)	0.0035 (5)
P2	0.0072 (6)	0.0076 (7)	0.0120 (7)	0.0015 (5)	0.0031 (5)	0.0042 (5)
P1	0.0068 (6)	0.0084 (7)	0.0103 (7)	0.0011 (5)	0.0032 (5)	0.0025 (5)
O2	0.0103 (18)	0.0121 (19)	0.0119 (19)	0.0050 (15)	0.0043 (15)	0.0042 (15)
O5	0.0077 (18)	0.011 (2)	0.019 (2)	0.0004 (15)	0.0019 (15)	0.0053 (16)
O3	0.0110 (18)	0.013 (2)	0.0126 (19)	−0.0004 (15)	0.0036 (15)	0.0006 (16)
O7	0.0103 (18)	0.0116 (19)	0.0136 (19)	0.0046 (15)	0.0047 (15)	0.0048 (15)
O9	0.029 (3)	0.024 (3)	0.021 (2)	0.004 (2)	0.014 (2)	0.007 (2)
O1	0.0082 (18)	0.0088 (19)	0.019 (2)	0.0013 (15)	0.0034 (16)	0.0043 (16)
C1	0.010 (2)	0.008 (2)	0.013 (3)	0.0014 (19)	0.003 (2)	0.003 (2)
O4	0.0085 (18)	0.0114 (19)	0.0153 (19)	0.0000 (14)	0.0039 (15)	0.0043 (15)
O8	0.018 (2)	0.026 (2)	0.013 (2)	−0.0028 (18)	0.0032 (17)	0.0052 (19)
N1	0.04 (2)	0.028 (18)	0.033 (19)	0.019 (13)	0.019 (16)	0.023 (13)
C4	0.026 (18)	0.019 (15)	0.018 (15)	−0.001 (12)	−0.008 (12)	0.008 (13)
C3	0.042 (5)	0.020 (3)	0.021 (3)	0.007 (3)	−0.004 (3)	0.006 (3)
C2	0.033 (4)	0.023 (4)	0.029 (4)	0.000 (3)	−0.008 (3)	0.009 (3)
O6	0.0148 (19)	0.0108 (19)	0.016 (2)	0.0016 (15)	0.0053 (16)	0.0073 (16)

### Geometric parameters (Å, °)

**Table:**

Cr1—O1	1.991 (4)	O1—H1B	0.83 (6)
Cr1—O2	1.956 (4)	O1—H1A	0.80 (6)
Cr1—O7	1.964 (4)	C1—H4	0.9700
P2—O6	1.499 (4)	C1—H5	0.9700
P2—O7	1.519 (4)	O8—H8	0.85 (7)
P2—O5	1.568 (4)	O8—H7	0.83 (6)
P2—C1	1.804 (6)	N1—C2	1.34 (5)
P1—O4	1.512 (4)	N1—C3	1.36 (4)
P1—O4	1.512 (4)	N1—H1	0.8600
P1—O2	1.515 (4)	C4—C3	1.40 (5)
P1—O3	1.568 (4)	C4—C2	1.41 (5)
P1—C1	1.797 (6)	C4—H4A	0.9300
O5—H6	0.83 (5)	C3—C2^i^	1.371 (13)
O3—H3	0.8200	C3—H3A	0.9300
O9—H9A	0.83 (6)	C2—C3^i^	1.371 (13)
O9—H10	0.84 (7)	C2—H2	0.9300
			
O2^ii^—Cr1—O2	180.00 (15)	P2—O5—H6	114 (5)
O2^ii^—Cr1—O7	88.35 (17)	P1—O3—H3	109.5
O2—Cr1—O7	91.65 (17)	P2—O7—Cr1	140.0 (3)
O2^ii^—Cr1—O7^ii^	91.65 (17)	H9A—O9—H10	108 (10)
O2—Cr1—O7^ii^	88.35 (17)	Cr1—O1—H1B	119 (6)
O7—Cr1—O7^ii^	180.0 (2)	Cr1—O1—H1A	120 (6)
O2^ii^—Cr1—O1^ii^	90.51 (17)	H1B—O1—H1A	107 (9)
O2—Cr1—O1^ii^	89.49 (17)	P1—C1—P2	114.8 (3)
O7—Cr1—O1^ii^	90.81 (18)	P1—C1—H4	108.6
O7^ii^—Cr1—O1^ii^	89.19 (18)	P2—C1—H4	108.6
O2^ii^—Cr1—O1	89.49 (17)	P1—C1—H5	108.6
O2—Cr1—O1	90.51 (17)	P2—C1—H5	108.6
O7—Cr1—O1	89.19 (18)	H4—C1—H5	107.5
O7^ii^—Cr1—O1	90.81 (18)	H8—O8—H7	114 (10)
O1^ii^—Cr1—O1	180.0 (3)	C2—N1—C3	123 (4)
O6—P2—O7	114.8 (2)	C2—N1—H1	118.3
O6—P2—O5	107.8 (2)	C3—N1—H1	118.3
O7—P2—O5	109.7 (2)	C3—C4—C2	116 (5)
O6—P2—C1	108.2 (3)	C3—C4—H4A	122.1
O7—P2—C1	109.0 (2)	C2—C4—H4A	122.1
O5—P2—C1	107.1 (2)	N1—C3—C2^i^	118 (2)
O4—P1—O2	115.5 (2)	C2^i^—C3—C4	122 (3)
O4—P1—O2	115.5 (2)	N1—C3—H3A	121.1
O4—P1—O3	107.9 (2)	C2^i^—C3—H3A	121.1
O4—P1—O3	107.9 (2)	C4—C3—H3A	116.7
O2—P1—O3	108.6 (2)	N1—C2—C3^i^	119 (2)
O4—P1—C1	110.1 (2)	C3^i^—C2—C4	122 (2)
O4—P1—C1	110.1 (2)	N1—C2—H2	120.7
O2—P1—C1	107.6 (2)	C3^i^—C2—H2	120.7
O3—P1—C1	106.9 (3)	C4—C2—H2	117.1
P1—O2—Cr1	134.1 (2)		

Symmetry codes:  (i) −*x*+1, −*y*+1, −*z*; (ii) −*x*+1, −*y*+1, −*z*+1.

### Hydrogen-bond geometry (Å, °)

**Table:**

*D*—H···*A*	*D*—H	H···*A*	*D*···*A*	*D*—H···*A*
C1—H4···O6^iii^	0.97	2.49	3.346 (7)	147
C4—H4A···O9^iv^	0.93	2.16	2.93 (7)	140
N1—H1···O9^iv^	0.86	2.32	3.03 (5)	141
O1—H1A···O6^iii^	0.80 (6)	1.83 (6)	2.634 (6)	176 (9)
O1—H1B···O4^v^	0.83 (6)	1.87 (6)	2.704 (6)	177 (9)
O3—H3···O8^vi^	0.82	1.83	2.629 (6)	163
O5—H6···O4^iv^	0.83 (5)	1.80 (5)	2.619 (6)	175 (10)
O8—H7···O6^vii^	0.83 (6)	1.86 (6)	2.687 (6)	171 (9)
O8—H8···O9	0.85 (7)	1.94 (8)	2.748 (7)	158 (11)
O9—H9A···O4	0.83 (6)	2.00 (6)	2.833 (6)	179 (10)
O9—H10···O8^viii^	0.84 (7)	1.99 (7)	2.820 (7)	174 (13)

Symmetry codes: (iii) −*x*+1, −*y*+2, −*z*+1; (iv) −*x*, −*y*+1, −*z*+1; (v) *x*+1, *y*, *z*; (vi) −*x*+1, −*y*+2, −*z*+2; (vii) *x*, *y*, *z*+1;  (viii) −*x*, −*y*+2, −*z*+2.
